# Microcystin-LR (MC-LR) Triggers Inflammatory Responses in Macrophages

**DOI:** 10.3390/ijms22189939

**Published:** 2021-09-14

**Authors:** Robin C. Su, Joshua D. Breidenbach, Khaled Alganem, Fatimah K. Khalaf, Benjamin W. French, Prabhatchandra Dube, Deepak Malhotra, Robert McCullumsmith, John B. Presloid, R. Mark Wooten, David J. Kennedy, Steven T. Haller

**Affiliations:** 1 Department of Medicine, The University of Toledo College of Medicine and Life Sciences, Toledo, OH 43614, USA; Robin.Su@rockets.utoledo.edu (R.C.S.); Joshua.Breidenbach@rockets.utoledo.edu (J.D.B.); Kareem.Khalaf@rockets.utoledo.edu (F.K.K.); Benjamin.French2@rockets.utoledo.edu (B.W.F.); prabhatchandra.dube@utoledo.edu (P.D.); Deepak.Malhotra@utoledo.edu (D.M.); 2 Department of Neuroscience, The University of Toledo College of Medicine and Life Sciences, Toledo, OH 43614, USA; Khaled.Alganem@rockets.utoledo.edu (K.A.); Robert.McCullumsmith@utoledo.edu (R.M.); 3 Neurosciences Center, Promedica, Toledo, OH 43614, USA; 4 Department of Medical Microbiology and Immunology, The University of Toledo College of Medicine and Life Sciences, Toledo, OH 43614, USA; john.presloid@utoledo.edu (J.B.P.); r.mark.wooten@utoledo.edu (R.M.W.)

**Keywords:** microcystin, colitis, macrophages

## Abstract

We were the first to previously report that microcystin-LR (MC-LR) has limited effects within the colons of healthy mice but has toxic effects within colons of mice with pre-existing inflammatory bowel disease. In the current investigation, we aimed to elucidate the mechanism by which MC-LR exacerbates colitis and to identify effective therapeutic targets. Through our current investigation, we report that there is a significantly greater recruitment of macrophages into colonic tissue with pre-existing colitis in the presence of MC-LR than in the absence of MC-LR. This is seen quantitatively through IHC staining and the enumeration of F4/80-positive macrophages and through gene expression analysis for *Cd68*, *Cd11b*, and *Cd163*. Exposure of isolated macrophages to MC-LR was found to directly upregulate macrophage activation markers *Tnf* and *Il1b*. Through a high-throughput, unbiased kinase activity profiling strategy, MC-LR-induced phosphorylation events were compared with potential inhibitors, and doramapimod was found to effectively prevent MC-LR-induced inflammatory responses in macrophages.

## 1. Introduction

Harmful algal blooms have quickly become a global health concern, appearing in freshwater environments around the world each year [1]. These blooms, which are an overgrowth of cyanobacteria, are capable of producing cyanotoxins such as Microcystins, of which, microcystin-LR (MC-LR) is one of the most frequently produced and one of the most toxic forms [2]. MC-LR has been well documented and extensively studied for its hepatotoxic effects [2,3,4,5,6,7,8]. Comparatively, little is known about the effects of MC-LR within other organ systems, such as the GI tract. We were the first to report that MC-LR has minimal GI effects in healthy mice, but significant GI toxicity in mice with pre-existing colitis [9]. Dextran sodium sulfate (DSS) can be used in mice to model colitis. DSS modeling is achieved by administering DSS via drinking water, and is capable of mimicking both acute and chronic colitis [10]. In either case, the colitis is a result of damage leading to significant changes in the large intestine and including modification of the gut microbiome. It has also been shown that some ingested DSS undergoes phagocytosis by macrophages along the intestinal lining, indicating macrophage activation in response to DSS exposure [11]. Importantly, macrophages have been shown to drive the disease pathology of inflammatory bowel disease and colitis [12]. Mice with pre-existing dextran sulfate sodium (DSS)-induced colitis that were also exposed to MC-LR experienced significant and prolonged body weight loss, the prolonged presence of blood within their stool, increased spleen weight as a gross indicator of inflammation, significantly greater colonic shortening and ulceration, and significantly elevated gene expression of the inflammatory markers *Tnf* and *Il1b* as compared with mice with colitis alone [9]. These novel findings suggested that whereas those with a healthy GI background do not experience major toxicity from MC-LR exposure, those with pre-existing GI conditions are more vulnerable and susceptible to MC-LR toxicity and are prone to a worsened overall disease state upon MC-LR exposure. Another consideration in the severity of colitis is TLR2. Normal TLR2 activity helps maintain intestinal epithelial structure and function in colitis models, reducing the damage done to the mucosal membrane in DSS-induced colitis. Additionally, studies have shown that the knockout and polymorphic loss of function of TLR2 results in a more severe presentation of colitis in animal models and ulcerative colitis patients, respectively [13,14]. Finally, prior work found that TLR2 may mediate the cellular response to MC-LR [15,16].

The aim of this study was to identify whether macrophages are found in greater quantity in the presence of MC-LR in the GI, which would suggest that MC-LR-driven recruitment of these inflammatory cells plays a key role in perpetuating pre-existing colitis. Separately, we sought to further confirm MC-LR’s capacity to elicit inflammatory responses from these cells, and subsequently use a high-throughput, unbiased approach to identify a therapeutic method of inhibiting this inflammatory response to MC-LR. Through the identification of a successful therapeutic measure, we believe this to be a significant milestone in identifying ways to protect more vulnerable populations with pre-existing colitis from the toxic effects of MC-LR.

## 2. Results

### 2.1. Characterization of Inflammatory Cell Infiltration of the Colon

**Figure 1:**
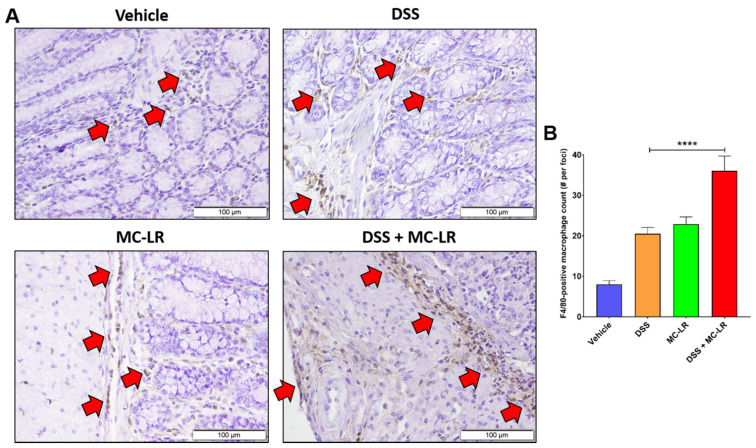
F4/80-positive macrophages in FFPE colonic sections of DSS-induced colitis model C57BL/6J mice. (**A**) IHC staining in: (Vehicle) control animals without DSS-induced colitis or MC-LR exposure. (DSS) DSS-induced colitis without MC-LR exposure. (MC-LR) MC-LR exposed animals without DSS-induced colitis. (DSS+MC-LR) DSS-induced colitis with MC-LR exposure. Red arrows denote positive F4/80 staining of macrophages. (**B**) Quantification of F4/80-positive macrophages by count in 10 random foci per animal (n = 3). Significance by one-way ANOVA (*p* < 0.0001) and **** = *p* < 0.0001 by Tukey’s multiple comparisons test.

We have previously shown that MC-LR has limited effects within the GI of healthy C57BL/6J mice, but has toxic effects in mice with pre-existing DSS-induced colitis (DSS+MC-LR) [9]. In mice with pre-existing colitis, MC-LR exposure prolonged weight loss and the presence of bloody stools, and increased spleen weight, colonic shortening, ulceration, and inflammation [9]. Hematoxylin and eosin (H&E) staining of formalin-fixed paraffin-embedded (FFPE) colonic sections revealed large numbers of inflammatory cell infiltrates in DSS mice, with increased infiltrates in DSS+MC-LR mice [9]. To further characterize this inflammation, exposure experiments were repeated and immunohistochemical (IHC) staining for F4/80-positive macrophages was completed (Figure 1). F4/80 is a widely used marker for mouse macrophages and has been used in over one hundred publications to date [17]. F4/80-positive macrophages were counted in 10 random foci per animal with 3 animals per group, revealing increased positive staining in DSS+MC-LR mouse colons compared with DSS mouse colons, demonstrated by the increase in brown 3,3′-Diaminobenzidine (DAB) staining (Figure 1), quantified in Figure 1B.

**Figure 2:**
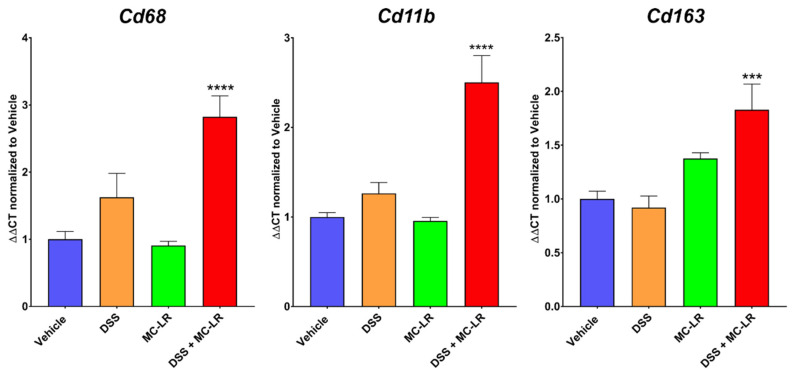
RT-PCR analysis for macrophage markers *Cd68*, *Cd11b*, and *Cd163* in colonic tissue from C57BL/6J mice. All values are normalized to housekeeping gene *18s* and presented as the mean fold change relative to Vehicle healthy mice ± SEM (n = 6–10 mice per group). *** *p* < 0.001 and **** *p* < 0.0001 vs. the control Vehicle group by one-way ANOVA with Tukey’s multiple comparisons.

To emphasize the differential abundance of macrophages in the colons of these mice, gene expression levels in colon tissues for macrophage markers Cd68, Cd11b, and Cd163 were determined by RT-PCR. This analysis revealed significantly upregulated expression in the colons of DSS+MC-LR mice compared with Vehicle control mice (Figure 2).

### 2.2. MC-LR Effects in Macrophages

**Figure 3:**
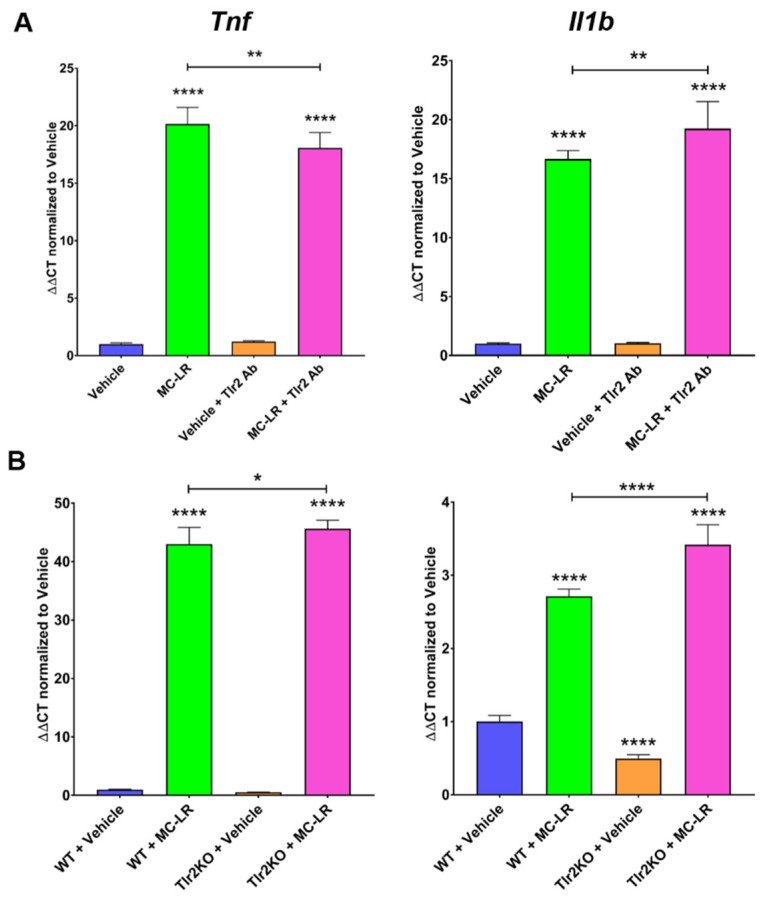
RT-PCR analysis for inflammatory markers *Tnf* and *Il1b* in ex vivo intraperitoneal macrophages. (**A**) Exposure of Dahl-S rat IP macrophages to Vehicle or MC-LR with or without anti-Tlr2 mAb pretreatment. (**B**) Exposure of C57BL/6J (WT) or Tlr2KO mouse IP macrophages to vehicle or MC-LR. All values are normalized to housekeeping gene 18S and presented as the mean fold change relative to Vehicle (**A**) or WT Vehicle (**B**) ± SEM (n = 3 samples per group). * *p* < 0.05, ** *p* < 0.01, and **** *p* < 0.0001 by one-way ANOVA with Tukey’s multiple comparisons.

In an attempt to elucidate the mechanism behind the apparent differences in macrophage abundance, we hypothesized that macrophages, initially recruited in response to either MC-LR or DSS, would become activated by the presence of MC-LR and produce cytokines and chemokines, triggering further macrophage recruitment. To test this, intraperitoneal (IP) macrophages were isolated from Dahl-S (S) rats and exposed to 10 μM MC-LR for 24 h. MC-LR induced significant increases in the expression of macrophage activation markers Tnf and Il1b as compared with control macrophages without MC-LR exposure (Figure 3A).

Exposure to MC-LR elicits an inflammatory response, and it has been suggested by Adamovsky et al. and Lin et al. that TLR2 may play a role in mediating this effect (20, 21). Therefore, we hypothesized that TLR2 would be required for the apparent MC-LR-mediated ex vivo macrophage activation. Pre-treatment of macrophages with 2.5 μg/mL anti-Tlr2 monoclonal antibody (mabg-mtlr2; Invivogen, San Diego, CA, USA) before MC-LR exposure led to a decrease in Tnf gene expression and an increase in Il1b gene expression as compared with macrophages exposed to MC-LR alone (Figure 3A). Separately, IP macrophages were isolated from C57BL/6J (WT) and Tlr2-knockout mice on the C57BL/6J background (Tlr2KO) mice and exposed to 10 μM MC-LR. As in the rat IP macrophages, MC-LR induced significant increases in the gene expression of Tnf and Il1b as compared with control macrophages (Figure 3B). Exposure of Tlr2KO IP macrophages to MC-LR led to an increase in Tnf gene expression and an increase in Il1b gene expression as compared with WT macrophages exposed to MC-LR (Figure 3B).

### 2.3. MC-LR Induced Macrophage Kinomics

**Figure 4:**
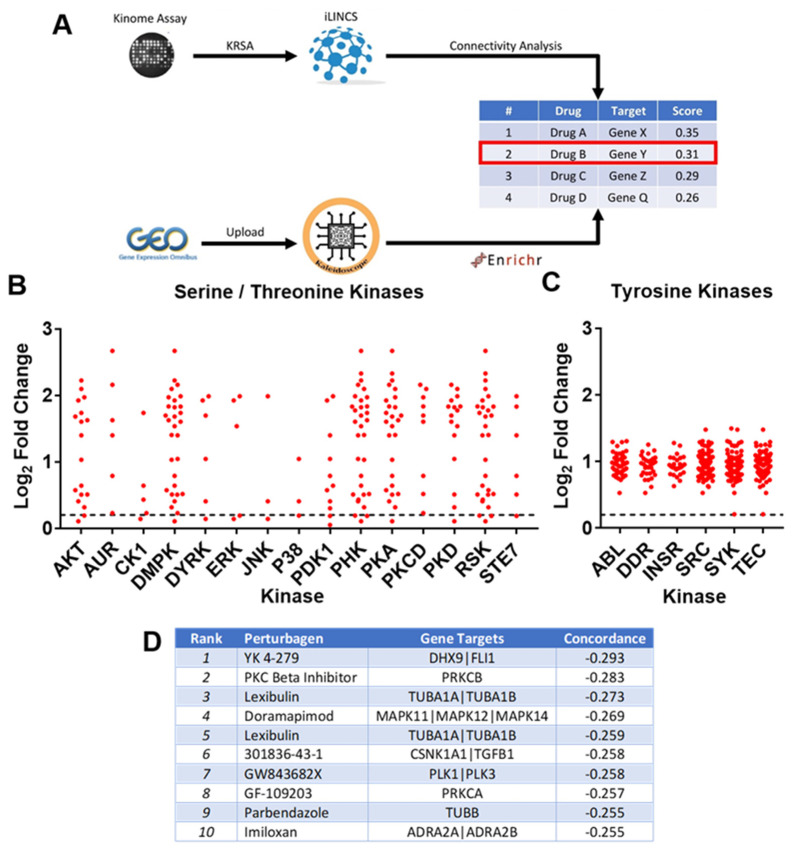
Kinome profiling and in silico workflow for the identification of MC-LR-induced kinase activity and potential inhibitory compounds. (**A**) Schematic summarizing the overall workflow. Gene expression profiles derived from kinome profiles and published MC-LR exposure gene expression profiles were compared against perturbagen signatures in iLINCS to generate a list of hypothetical inhibitory compounds for the MC-LR-induced kinase activity. (**B**) Kinase activity from the serine/threonine kinase (STK) (**C**) and tyrosine kinase (PTK) arrays. (**D**) Identified hypothetical inhibitory compounds ranked by their inverse concordance with the MC-LR-induced signatures.

To further dissect the macrophage-activating effect of MC-LR, isolated rat IP macrophages were exposed to 10 μM MC-LR and peptide phosphorylation microarray data were generated using the Pamstation12 (PamGene International, The Netherlands) kinome profiling system (Figure 4). Specifically, the activities of serine/threonine kinases (STK) and tyrosine kinases (PTK) were assessed. 

In order to identify peptides with robust changes in magnitude of phosphorylation, a log2-fold change threshold cutoff was set at |log2FC| ≥ 0.2. The profile of differentially phosphorylated peptides was used to approximate upstream kinase activity through in silico phosphosite-substrate databases. Comparing observed peptide/kinase matches with a random sampling analysis revealed that kinases increased activity (Figure 4B,C).

All altered kinases were upregulated; therefore, a “consensus gene expression signature” was constructed by gathering existing expression signatures from over-expression experiments in the integrative Library of Integrated Network-based Cellular Signatures (iLINCS) system and averaging all profiles. We then interrogated the iLINCS system for perturbagen signatures which were inversely correlated with the expression of our consensus gene expression signature (negative concordance score) (Figure 4D), which would hypothetically reverse the effects of the MC-LR-induced kinase activity. This provided us with a list of compounds that putatively reverse the effects of MC-LR.

To enrich our list of candidate compounds that reverse the effects of MC-LR, we took advantage of gene expression datasets from four published microcystin studies sourced from the NCBI Gene Expression Omnibus (GEO) (GSE59495, Walker 2014; GSE59906, Auerbach 2014; GSE12214, Rogers 2009; GSE29861, Zeller 2012). These datasets were processed and analyzed using GEO2R, Kaleidoscope and Enrichr in order to profile common differentially expressed genes. This enrichment analysis identified target pathways shared with the kinome analysis. Of particular interest were the MAPK signaling pathways, which were found to be most common amongst the differentially expressed genes. The differential expression of MAPK genes correlates with the previously identified inhibitory compound, doramapimod’s pathway of action (Figure 4D).

### 2.4. Doramapimod’s Effects on Macrophage Inflammatory Responses to MC-LR

**Figure 5:**
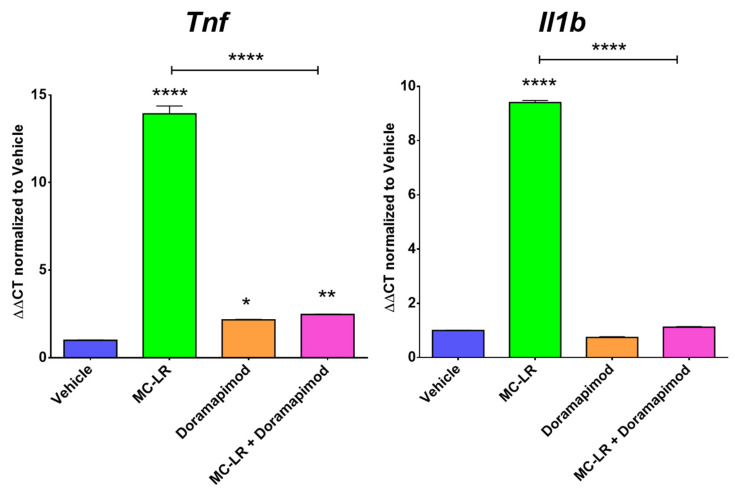
RT-PCR analysis for inflammatory markers *Tnf* and *Il1b* in ex vivo intraperitoneal macrophages after doramapimod pretreatment. All values are normalized to housekeeping gene 18S and presented as the mean fold change relative to vehicle control ± SEM (n = 3 samples per group). * *p* < 0.05, ** *p* < 0.01, and **** *p* < 0.0001 by one-way ANOVA with Tukey’s multiple comparisons.

The compound doramapimod, as identified through kinase profiling and GEO signatures (Figure 4), was used to treat rat IP macrophages exposed to MC-LR. Importantly, pretreatment with 10 μM doramapimod followed by MC-LR exposure significantly inhibited MC-LR’s ability to induce increased *Tnf* expression, and completely inhibited *Il1b* expression in macrophages (Figure 5).

## 3. Discussion

We have previously shown that MC-LR has limited effects within healthy colons but exacerbates the overall disease state within colons with pre-existing colitis [9]. The current study is the first to identify macrophages as an important mechanistic contributor in MC-LR-mediated colitis exacerbation. We observed that, amidst large inflammatory cell infiltration into colonic tissue, macrophages are present in DSS-induced colitis and their levels are elevated within colons with colitis and additional MC-LR exposure. We have shown this through IHC staining for F4/80-positive macrophages and quantitative measurements of Cd68, Cd11b, and Cd163 expression, which are highly expressed on macrophages [18,19,20,21,22,23,24,25,26,27]. In vitro, we also observed that MC-LR induces large inflammatory responses by macrophages, by stimulating the upregulation of *Tnf* and *Il1b*, which likely plays a key role in driving the enhanced disease state seen in MC-LR-exposed mice with pre-existing colitis, given that *Tnf* and *Il1b* are also upregulated in vivo. Our goal was to identify therapeutic methods for preventing MC-LR-mediated inflammatory responses in macrophages.

Recent studies have reported on MC-LR’s tendency to stimulate strong inflammatory responses within zebrafish spleens and murine RAW 264.7 cells, which is an Abelson murine leukemia virus-transformed macrophage cell line [15,16,28]. Similar to the results we observed, Adamovsky et al. and Lin et al. reported that the end product of MC-LR exposure is the upregulation of inflammatory mediators, such as *Tnf* and *Il1b* [15,16]. Although the mechanism remains unclear, Adamovsky et al. and Lin et al. have previously suggested that TLRs may play a role in mediating MC-LR’s effects, specifically, TLR2 [15,16]. Given that TLR2 has not previously been investigated, we first investigated whether TLR2 is involved in stimulating MC-LR-mediated inflammatory responses in macrophages. We utilized a Tlr2-inhibiting antibody in the presence of MC-LR exposure. Ant-Tlr2 mAb pretreatment only minimally decreased MC-LR-induced *Tnf* upregulation and increased MC-LR-induced *Il1b* upregulation. To further investigate these effects, we also utilized Tlr2KO macrophages. Knocking out Tlr2 was found to further increase MC-LR-induced *Tnf* upregulation and *Il1b* upregulation. In our hands, blocking Tlr2 failed to produce a consistent and robust inhibitory effect on macrophage inflammatory responses to MC-LR exposure. Specifically, Tlr2 inhibition by monoclonal antibody resulted in a decrease in the relative expression of Tnf (from 20.14 +/− 0.59 to 18.05 +/− 0.56) and an increase in the relative expression of Il1b (from 16.66 +/− 0.29 to 19.23 +/− 0.94). Furthermore, exposure of macrophages from Tlr2KO animals resulted in an increase in the relative expression of Tnf (from 43.01 +/− 1.15 to 45.61 +/− 0.60) and of Il1b (from 2.71 +/− 0.04 to 3.42 +/− 0.11). The direction of change after Tlr2 inhibition was more often an increase in the response rather than a decrease, which suggests that MC-LR-induced macrophage activation occurs at least partially through a mechanism other than Tlr2. We subsequently aimed to utilize a high-throughput, unbiased approach to identify specific kinome profiles involved in MC-LR-induced inflammatory cytokine upregulation in macrophages, and identify inhibitors that could specifically target those signatures. This analysis suggested that the MAPK inhibitor doramapimod may be able to counteract the differential kinase activity from MC-LR exposure in macrophages. Doramapimod pretreatment was able to completely inhibit MC-LR’s ability to induce *Il1b* gene expression and significantly inhibit *Tnf* gene expression in macrophages.

It is important to note that all measurements of Tnf and Il1b in this study are referring to gene expression by RT-PCR, and measurements of secreted Tnf and Il1b protein would strengthen our investigation. Although there was an apparent inhibition of MC-LR-induced pro-inflammatory gene expression by doramapimod, this effect is not specific for MC-LR-induced inflammation, because doramapimod is a known anti-inflammatory compound [29,30]. Likewise, other common compounds used for their anti-inflammatory properties may be useful in the inhibition of MC-LR-induced inflammation. Nevertheless, our study provides a rational approach and methodology by which pharmacologic agents that attenuate the inflammatory effects of toxins such as MC-LR can be identified and repurposed according to their kinomic signature.

We conclude that doramapimod is an effective therapeutic agent in reversing the inflammatory responses of macrophages to MC-LR exposure and could potentially serve as a preventative or therapeutic tool in populations with pre-existing colitis, which are more vulnerable and susceptible to the toxic effects of MC-LR.

## 4. Materials and Methods

### 4.1. Animal Studies

All animal experimentation was conducted in accordance with the National Institutes of Health (NIH) Guide for the Care and Use of Laboratory Animals under protocols approved by The University of Toledo Institutional Animal Care and Use Committee (IACUC protocol #108663, approval date 9 February 2016). All animals were housed in a specific-pathogen-free facility, maintained at standard conditions of 23 ± 1 °C under a 12 h light cycle and were allowed to eat a normal chow diet ad libitum. The DSS-induced colitis model and MC-LR exposures were conducted as previously described [9]. Briefly, 8-week-old male C57BL/6J mice (Jackson Laboratory) were given either water or 3% DSS in water ad libitum for 7 days. Then, mice were given water or 1000 μg/kg MC-LR (item no. 10007188; Cayman Chemical, Ann Arbor, MI, USA) daily for 7 days by oral gavage. This yielded 4 groups: Water-only (Vehicle), n = 6; MC-LR-only (MC-LR), n = 10; DSS-only (DSS), n = 6; and MC-LR following DSS (DSS+MC-LR), n = 10. After euthanasia on day 14 of the study, colonic tissue was harvested and flash-frozen in liquid nitrogen for RT-PCR analysis. Remaining colonic tissues were cut longitudinally, wrapped, and placed in cassettes for fixation in 10% neutral buffered formalin. Immunohistochemistry staining of formalin-fixed paraffin-embedded tissues (FFPE) was performed as described by the primary antibody manufacturer using anti-F4/80 mAb (Cl:A3-1; Bio-Rad, Hercules, CA, USA) at a 1:100 dilution. Images were collected at 40X and F4/80-positive macrophages were counted in 10 random foci from each animal (n = 3).

### 4.2. In Vitro Macrophage Experiments

Male dahl-S rats (S), male C57BL/6J mice, or male Tlr2 knockout mice (B6.129-Tlr2^tm1Kir^/J; Jackson Laboratory, Bar Harbor, ME, USA) were injected intraperitoneally with thioglycolate, as previously described [31]. After 72 h, peritoneal macrophages were obtained by lavage and adherent macrophages were allowed to settle for another 72 h. Cells were allowed to grow in 12-well plates and were cultured in Dulbecco’s modified Eagle’s medium (DMEM) (Catalog No. 11995065; ThermoFisher Scientific, Waltham, MA, USA) supplemented with 10% fetal bovine serum (Rocky Mountain Biologicals, Inc, Missoula, MT, USA) and 1% penicillin–streptomycin solution (Caisson Labs, Smithfield, UT, USA).

MC-LR was used at a dose of 10 μM. Anti-Tlr2 monoclonal Ab (Item No. mabg-mtlr2; InvivoGen, San Diego, CA, USA), used at a dose of 2.5 μg/mL, with pretreatments for 1 h. Doramapimod (Item No. 10460; Cayman Chemical, Ann Arbor, MI, USA), used at a dose of 10 μM, with pretreatments for 30 min. All treatments were for 24 h in duration. Treatments were preceded by 24 h of serum starvation using DMEM supplemented with only 1% penicillin–streptomycin solution, and treatments were prepared in the same serum-starved conditions.

### 4.3. RNA Extraction and RT-PCR Method

RNA was isolated utilizing the Qiagen RNeasy Plus Mini Kit (Catalog No. 74134; Qiagen, Germantown, MD, USA) and the Qiagen QIAcube extraction methodology. Approximately 500 ng of extracted RNA was used to synthesize cDNA (QIAGEN’s RT2 First Strand Kit, Catalog No. 330401). RT-PCR was performed utilizing QIAGEN’s Rotor-Gene Q thermo-cycler. Calculation of gene expression was conducted by comparing the relative change in cycle threshold value (ΔCt). Fold change in expression was calculated using the 2-ΔΔCt equation, as previously described [32]. The following rat Taqman primers were used and obtained from Thermo Fisher Scientific (Waltham, MA, USA): Tnf (Rn99999017_m1) and Il1b (Rn00580432_m1). The following mouse Taqman primers were used and obtained from Thermo Fisher Scientific: Tnf (Mm00443258_m1), Il1b (Mm00434228_m1), Cd68 (Mm03047343_m1), Cd11b (Mm00434455_m1), and Cd163 (Mm00474091_m1). For the normalization of transcript expression, 18s rRNA from Thermo Fisher Scientific was used as a housekeeping gene (catalog no. 4319413E).

### 4.4. Kinase Activity Profiling

Utilizing a methodology that we have applied previously, profiling of serine/threonine and tyrosine kinase activity was performed following the generation of peptide phosphorylation array data using Pamstation12 (PamGene International, s-Hertogenbosch, The Netherlands) and serine/threonine kinase (STK) and tyrosine kinase (PTK) PamChips [33,34,35]. The device measures the degree of the phosphorylation in real time by detecting fluorescently labeled antibodies at five different exposure times (10, 20, 50, 100, 200 ms) using Evolve (PamGene) kinetic image capture software. The BioNavigator software (PamGene) was used to convert the images into numerical values for extended analyses. The numbers produced by this software represent the median value of the foreground pixels minus the median value of the background pixels to produce the median signal minus the background. For each peptide and based on the signal intensity values relative to the five different exposure times, a linear regression slope was calculated, scaled by multiplying it by 100, and log-transformed to represent the final signal. Using these signal values, log base twofold changes (Log2FC) of signal intensities between the sample groups for each peptide were calculated. After filtering out peptides with very low signals and/or non-linear slopes, a Log2FC threshold cutoff was set (|log2FC| ≥ 0.2) to represent meaningful changes in the degree of phosphorylation of peptides between the compared groups. Using the list of peptides with differential phosphorylation levels, an upstream kinase analysis was performed. Upstream kinase mapping was performed by utilizing the in silico phosphosite-substrate databases GPS 3.0, Kinexus Phosphonet (http://www.phosphonet.ca/, accessed on 15 November 2019), PhosphoELM (http://phospho.elm.eu.org/ (accessed on 15 November 2019)), and PhosphoSite Plus (www.phosphosite.org, accessed on 15 November 2019) [36,37]. To determine which kinases were most likely to be implicated in our experiment, a random sampling analysis was performed using the Kinome Random Sampling Analyzer (KRSA) [38]. This analysis randomly selected the same number of the set of differentially phosphorylated peptides from the chip 2000 times. Kinases predicted to target each phosphorylation site were identified and the frequency of each kinase was calculated using all of the 2000 permutated datasets. From these datasets, an expected distribution for each kinase with their means and standard deviations were derived. Kinases with observed frequencies falling outside two standard deviations from the expected mean (generated from the permutation datasets) were considered as “hits”. Using both the STK and PTK chips, 22 kinase hits were identified.

### 4.5. In Silico Drug Repurposing Analysis

The Library of Integrated Network-Based Cellular Signatures (LINCS) is a large multi-omics profiling database. For transcriptional profiling, it utilizes the L1000 assay, which is a gene-expression profiling assay based on the direct measurement of a reduced representation of the transcriptome under different perturbations: gene knockdown, gene overexpression, or drug treatment [39]. The Integrative LINCS (iLINCS) is a web platform for the analysis of LINCS datasets that was developed under the LINCS consortium (www.ilincs.org, accessed on 15 November 2019). iLINCS uses weighted Pearson correlation analysis to measure the concordance between signatures [40].

All peptides that were mapped to our kinase hits had a higher degree of phosphorylation compared to the control samples, we queried LINCS gene over-expression signatures of our kinase hit genes. Mapping the kinase hits to their corresponding genes resulted in a list of 37 genes. Given that the LINCS database contains profiling signatures of different cell lines, we limited our query to just the A375 cell line because it had the most gene signatures in common with our targets; thus, we aimed to capture as many of our targets as possible available in the database. We narrowed down our search to a single cell line specifically to limit cell line signature variation. A consensus signature was generated by calculating the mean of the collected signatures (14 gene overexpression signatures). Then, iLINCS was used to query for signatures that can “reverse” our combined signature. We ranked the resulting query based on their concordance scores relative to our combined signature. Again, only A375 signatures were selected, and a set of candidate drugs were identified that negatively correlated with our combined signature.

### 4.6. Dataset Pathway Analysis

Gene expression datasets were obtained from NCBI Gene Expression Omnibus (GEO) and analyzed with GEO2R [41]. These datasets were uploaded to Kaleidoscope [42]. Common differentially expressed genes across all datasets were identified. Genes were ranked based on log2-fold change values. The top 100 hits were analyzed for signaling pathways by Enrichr [43,44]).

### 4.7. Statistical Analysis

All RT-PCR data are presented as the mean ± SEM. Statistical analysis was conducted with GraphPad Prism 7.0d software (GraphPad Software, San Diego, CA, USA) using one-way ANOVA. Significance was determined if *p*-values were <0.05.

## Data Availability

All reported data are available via the corresponding author upon request.
